# Ameliorative Effects of *Prunella vulgaris* on Lower Urinary Tract Symptoms Induced by Benign Prostatic Hyperplasia in SD Rats via Nitric Oxide and Potassium Channels

**DOI:** 10.3390/ph18030400

**Published:** 2025-03-12

**Authors:** Beno Ramesh Nirujan, Jeongsook Kim, Eun-Bok Baek, Kyungmi Kim, Nishani Jayanika Jayathilake, Youn Gil Kwak, Mi Ran Jang, Hyo Seong Ji, Hyo-Jung Kwun, Kyu Pil Lee

**Affiliations:** 1 Department of Veterinary Physiology, College of Veterinary Medicine, Chungnam National University, Daejeon 34134, Republic of Koreajeongsook@o.cnu.ac.kr (J.K.); snowbells2@naver.com (K.K.); nishanijayanika1@gmail.com (N.J.J.); 2 Department of Veterinary Pathology, College of Veterinary Medicine, Chungnam National University, Daejeon 34134, Republic of Korea; baekeunbok@hanmail.net (E.-B.B.); hyojung@cnu.ac.kr (H.-J.K.); 3 Huons Foodience Co., Ltd., Geumsan 32724, Republic of Korea; kyg@huonsfoodience.com (Y.G.K.); rose86mr@huonsfoodience.com (M.R.J.); hsji@huonsfoodience.com (H.S.J.)

**Keywords:** *Prunella vulgaris* (PV), benign prostatic hyperplasia (BPH), relaxation, voiding, efficacy

## Abstract

**Background:** Lower urinary tract symptoms (LUTS) due to prostate hyperplasia are the most frequent urological symptoms in elderly men. Current pharmacological treatments for LUTS and benign prostatic hyperplasia (BPH) are widely used in clinical practice; however, adverse effects associated with these drugs have been reported for sexual dysfunction and orthostatic hypotension. *Prunella vulgaris* (PV) is a medicinal herb that has a long history of use. This study aimed to address this gap by investigating the relaxant activity of PV extract (PVE) on rat prostate smooth muscle ex vivo and evaluating intravesical cystometry for its potential. **Methods and Results:** Ten male Sprague Dawley (SD) rats were used to study the relaxant efficacy of PVE and its constituents in isometric contraction ex vivo. Thirty-six SD rats were randomly assigned to six groups of six animals (n = 6) and administered testosterone propionate (TP; 3 mg/kg) daily for 4 weeks to induce BPH. Groups of BPH rats were treated with or without PVE (30, 60, or 90 mg/kg) via oral gavage. At the end of the experiments, the animals were subjected to intravesical pressure under urethane anesthesia. After successful cystometric recording, rats were euthanized with carbon dioxide. Prostate and bladder tissues were harvested and processed for histological and biochemical analysis. The results demonstrated that PVE exerted relaxant effects on prostatic smooth muscle in a concentration-dependent manner, mediated by nitric oxide and potassium channels, without antagonizing adrenergic receptors. Additionally, intravesical cystometry in SD rats treated with oral gavage of PVE for 4 weeks showed a significant improvement in voiding abnormalities. **Conclusions:** These findings suggest the potential of PV and its compounds as a therapeutic strategy to improve LUTS associated with BPH.

## 1. Introduction

Benign prostatic hyperplasia (BPH) is a prevalent urological condition in elderly men, with its occurrence being age-dependent, affecting approximately 50% of men by the age of 60 and 80% of men over age of 90 [1,2]. BPH is characterized by the non-malignant proliferation of stromal and epithelial cells in the prostatic transitional zone [3,4]. These changes lead to enlargement of prostatic nodules, fibrosis, and alterations in smooth muscle function, which may cause partial or complete urethral obstruction. Subsequent bladder outlet obstruction, along with increased bladder muscle tone and secondary detrusor dysfunction, contributes to the development of lower urinary tract symptoms (LUTS) [5]. LUTS encompass a range of symptoms, including urgency, frequency, nocturia, and urge incontinence [6]. Individuals with LUTS associated with BPH (LUTS/BPH) frequently seek medical intervention because the symptoms have a significant impact on their quality of life [7].

The precise molecular mechanism of BPH is not fully understood [8]. Prostate growth strongly relies on the conversion of testosterone to dihydrotestosterone (DHT) by 5α-reductase, followed by DHT-binding androgen receptors (AR) to stimulate protein synthesis and subsequent cell proliferation [4]. DHT concentration increases in BPH, correlating with enlarged prostate volume and higher prevalence of LUTS [9,10]. An enlarged prostate can lead to an increase in the static tone of the prostate smooth muscle surrounding the urethra, resulting in obstruction. Currently, pharmacological interventions to treat BPH/LUTS include 5α-reductase inhibitors, α1-blockers, antimuscarinics, and phosphodiesterase 5 inhibitors, which have shown efficacy in reducing prostate size and alleviating urethral obstruction by decreasing the tone of prostate smooth muscle [11,12]. However, the aforementioned medications can cause various adverse effects when taken for a long period of time [12]. In particular, adverse effects such as impotence, sexual dysfunction, cardiovascular side effects, urinary incontinence, headache, and indigestion have been reported [11]. Since BPH is often associated with cardiovascular risk factors such as age, hypertension, hyperlipidemia, and heart disease, the use of these medications requires careful consideration [13]. Therefore, phytotherapy is proposed as a potential alternative treatment for BPH/LUTS with good tolerability and minimal side effects [14].

*Prunella vulgaris* L. (PV) is a perennial herbaceous plant belonging to the *Lamiaceae* family, native to Europe and Asia [15,16]. Historically, it has been widely utilized in traditional medicine for the treatment of thyroid dysfunction, mastitis, pulmonary tuberculosis, infectious hepatitis, and arterial hypertension [15,17,18]. Additionally, PV is renowned for its diverse pharmacological properties, including antioxidant, anti-allergic, anti-inflammatory, and antimicrobial activities [15,17,19].

Based on previous studies, the primary bioactive compounds PV include polyphenols (flavonoids and phenolic acids), carbohydrates, and triterpenes [19,20,21]. Among these, ursolic and oleanolic acids, the major triterpenoids, exhibit anti-inflammatory, antioxidant, and antihypertensive properties [22,23]. PV ethanol extract contains abundant antioxidant polyphenols, including caffeic acid, rosmarinic acid, rutin, and quercetin [24]. In particular, quercetin and its derivatives have been reported to be effective in alleviating benign prostatic hyperplasia [25,26]. Additionally, caffeic and rosmarinic acids, essential phenolic acids, contribute to PV’s anti-inflammatory and antioxidant activities [27]. PV also contains fatty acids and volatile oils with reported biological functions [16].

Therefore, we hypothesized that PV may have therapeutic potential in mitigating BPH pathology with various biological activities. The fruit spike of the PV was extracted using maceration with an ethanol solvent to obtain the PV extract (PVE). Previous studies have shown that PVE extracted using the same ethanol maceration method alleviates prostate hyperplasia by regulating the proliferation and apoptosis of prostatic cells [28]. This study further investigated the relaxant effects of PVE and its potential bioactive constituents on ex vivo prostate smooth muscle strip tension. In addition, the effect of PVE on BPH/LUTS was investigated using a BPH-induced Sprague Dawley (SD) rat model, with a focus on intravesical pressure changes.

## 2. Results

### 2.1. PVE Extract Analysis

**Figure 1:**
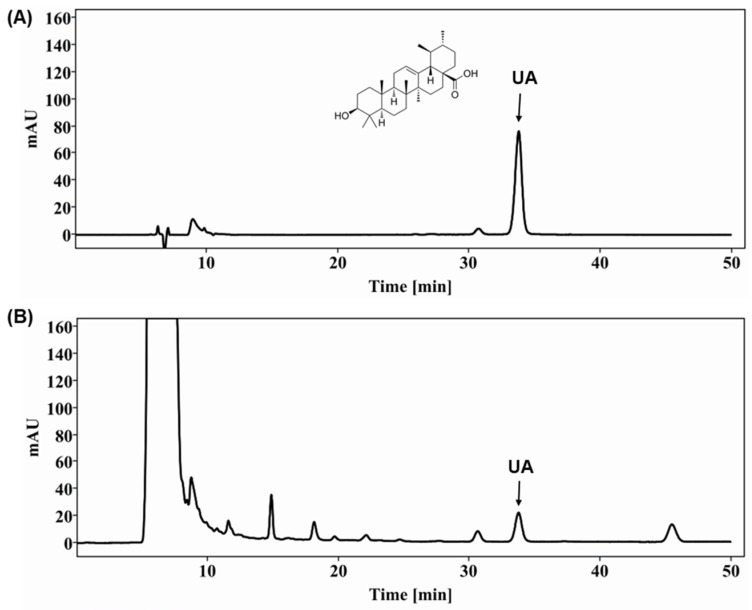
HPLC-UV chromatogram of (**A**) ursolic acid (UA) standard and (**B**) PVE. The detection wavelength was 210 nm.

We used the HPLC (high-performance liquid chromatography) technique to analyze PVE, and the results showed that ursolic acid (UA) is a major compound of PVE. Standard chemical, UA, was compared in terms of their retention times and spectrums with their values from the extract. The chromatograms for standards and PVE are shown in Figure 1. According to the HPLC results, quantitative analysis using standard chemicals confirmed that UA was contained in the PVE at a concentration of 7.14 mg/g.

### 2.2. PVE Attenuates Prostate Smooth Muscle Contraction Induced by PE

**Figure 2:**
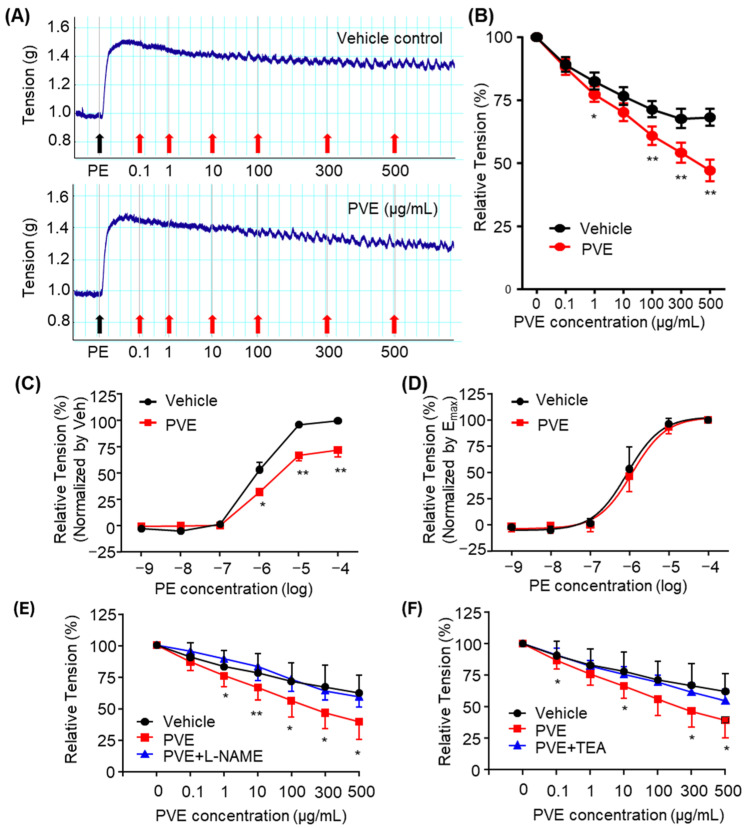
Relaxative effects of PVE on PE-induced contracted prostate smooth muscle strip from male SD rats. (**A**) Representative traces for treatment of vehicle (upper) and PVE (lower) on 10 μM phenylephrine (PE)-induced contraction of prostate smooth muscle. (**B**) The effects of PVE on PE-induced tone in prostate smooth muscle strips are summarized. (**C**,**D**) Dose-constriction responses to PE in the presence or absence of PVE are compared. (**C**) Preincubating at 300 ug/mL dramatically reduced the PE-induced contraction. (**D**) However, the dose–response curve was not changed in both conditions. (**E**,**F**) The relaxative effects of PVE were attenuated in the presence of L-NAME (nitric oxide synthase inhibitor) and TEA (tetraethylammonium, K^+^ channel inhibitor). Data are the mean ± SEM analyzed using a one-way ANOVA followed by a Tukey’s multiple comparison test; * and ** denote *p* < 0.05 and *p* < 0.01 compared with vehicle response at each concentration, respectively.

Phenylephrine (PE) is an α1-adrenergic receptor agonist and induces adrenergic contraction in prostate smooth muscle. Treatment with PVE significantly reduced the relative tension of prostate smooth muscle strips contracted by 10 μM PE compared to the vehicle control (Figure 2A,B). PVE concentrations of 1, 100, 300, and 500 μg/mL produced significant relaxation compared to vehicle control. To further evaluate whether PVE modulates PE-induced adrenergic prostate smooth muscle contraction, the strips were incubated with 300 μg/mL of PVE for 30 min, followed by measurement of contractile responses to increasing concentrations of PE. The results showed that PE-induced max contraction was inhibited in the PVE-treated group compared to the vehicle control (Figure 2C). However, the concentration–response curves of vehicle control and PVE pretreatment at 300 μg/mL, when normalized to maximal contraction, showed no significant differences (Figure 2D), suggesting that PVE does not act as a competitive antagonist to the α1-adrenergic receptor.

### 2.3. The Effect of PVE on Inhibiting PE-Induced Prostate Smooth Muscle Contraction Was Blocked by L-NAME and TEA

To determine the mechanisms underlying the relaxing effect of PVE on prostate smooth muscle, pre-contract with PE tissue strips were treated with PVE in the presence of L-NAME, a nitric oxide synthase (NOS) inhibitor, or TEA, a K^+^ channel inhibitor. Both L-NAME and TEA attenuated the relaxing effect of PVE on prostate smooth muscle. 100 μM L-NAME significantly reduced the relaxation induced by PVE at a concentration of 1, 10, 100, 300, and 500 μg/mL (Figure 2E), while 1 mM TEA inhibited PVE-mediated relaxation at 0.1, 10, 300, and 500 μg/mL (Figure 2F).

### 2.4. Quercetin Inhibits PE-Induced Prostate Smooth Muscle Contraction

**Figure 3:**
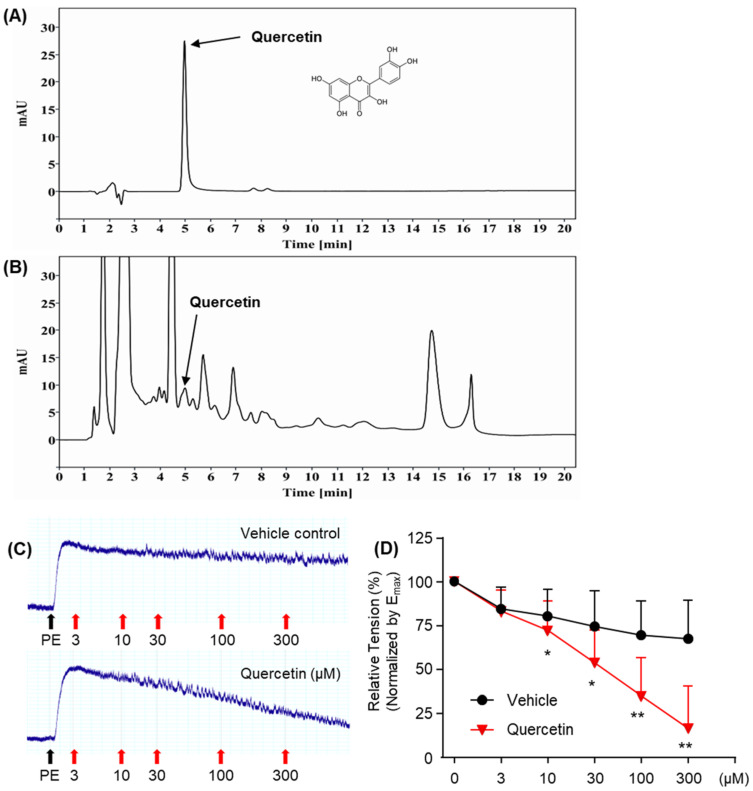
HPLC-UV chromatogram and relaxation effects on PE-induced prostate smooth muscle contraction of quercetin. HPLC-UV chromatogram of quercetin standard (**A**) and PVE (**B**). The detection wavelength was 210 nm. (**C**,**D**) Quercetin induced concentration-dependent relaxation of prostate smooth muscle contractions at concentrations ranging from 10–300 μM. Data are the mean ± SEM analyzed using a one-way ANOVA followed by a Tukey’s multiple comparison test; * *p* < 0.05 and ** *p* < 0.01 indicate significant differences compared to the DMSO vehicle response at each concentration.

PVE effectively relaxed PE-induced prostate smooth muscle contraction. However, UA, which was identified as the major compound in the previous experiment, only showed a relaxation effect of 7.175 ± 5.27% (n = 4) at a concentration of 50 μg/mL, which was not statistically significant. To identify the compound in PVE responsible for relaxant effects, quercetin, which has been previously found in PV [29], was tested on the relaxation of prostate smooth muscle. First, HPLC was used to qualitatively confirm the presence of quercetin in PVE used in this study. Quercetin was identified with a retention time of 4.9 min (Figure 3A,B). The effects of quercetin were examined by applying accumulatively increasing its concentrations in prostate smooth muscle pretreated with 30 μM PE, where it exhibited potent relaxation at concentrations above 30 μM (Figure 3C,D). 

### 2.5. PVE Administration in In Vivo TP-Induced BPH Model

**Figure 4:**
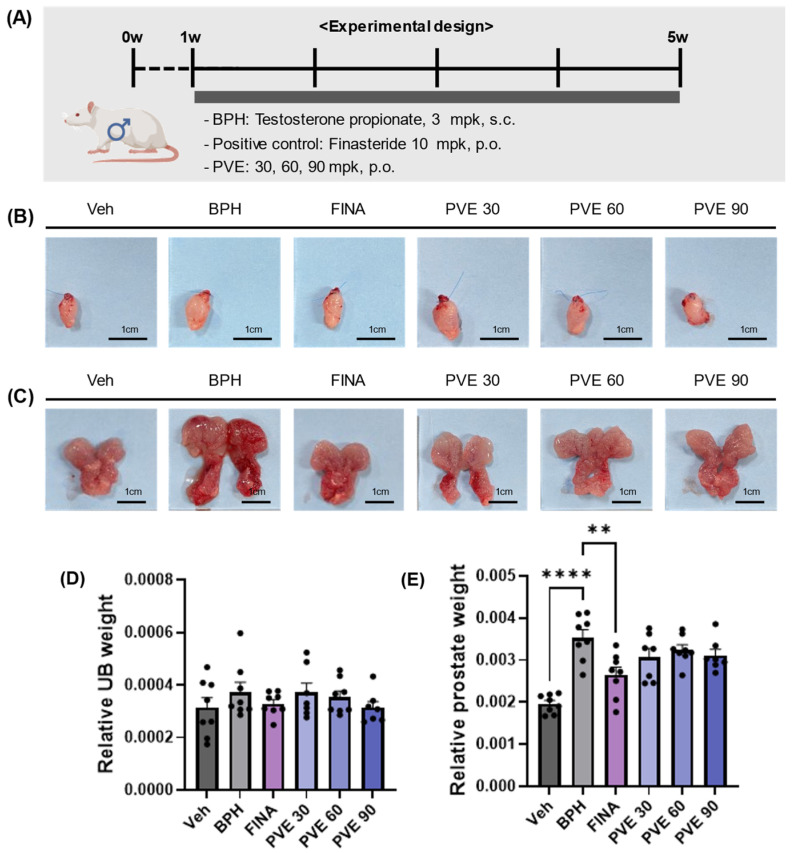
Effects of PVE on TP-induced BPH model animals. (**A**) Schematic representation of the experimental design. (**B**,**C**) Representative images of the urinary bladder and prostate tissues from each experimental group on Day 28. (**D**,**E**) Changes in the urinary bladder and prostate weight relative to body weight were analyzed across the Vehicle, BPH, Finasteride, PVE 30 mg/kg, PVE 60 mg/kg, and PVE 90 mg/kg groups. Data are shown as mean ± SEM. Statistical significance was evaluated using One-way ANOVA followed by Tukey’s multiple comparison test; ** *p* < 0.01 compared to the vehicle group, **** *p* < 0.0001 compared to the Finasteride group.

To investigate the effect of PVE in testosterone propionate (TP)-induced BPH model rats, the following in vivo experiments were conducted (Figure 4A). At the end of the treatment period, the rats were humanely euthanized, and their bladders and prostates were excised, photographed, and weighed. The weight of the bladder and prostate was measured in relation to the body weight of the rats. There was no significant difference in bladder weight between all groups (Figure 4B,D). However, in the BPH group, the prostates were notably enlarged, exhibited a reddish discoloration, and showed a significant increase in the relative prostate weight compared to the vehicle control (Figure 4C,E). The increased prostate weight seen in the BPH group was significantly reduced in the positive control finasteride group. PVE did not reduce the increase in prostate weight induced by TP at all concentrations.

### 2.6. PVE Alleviates Histologic Changes in the Prostate in In Vivo TP-Induced BPH Model

**Figure 5:**
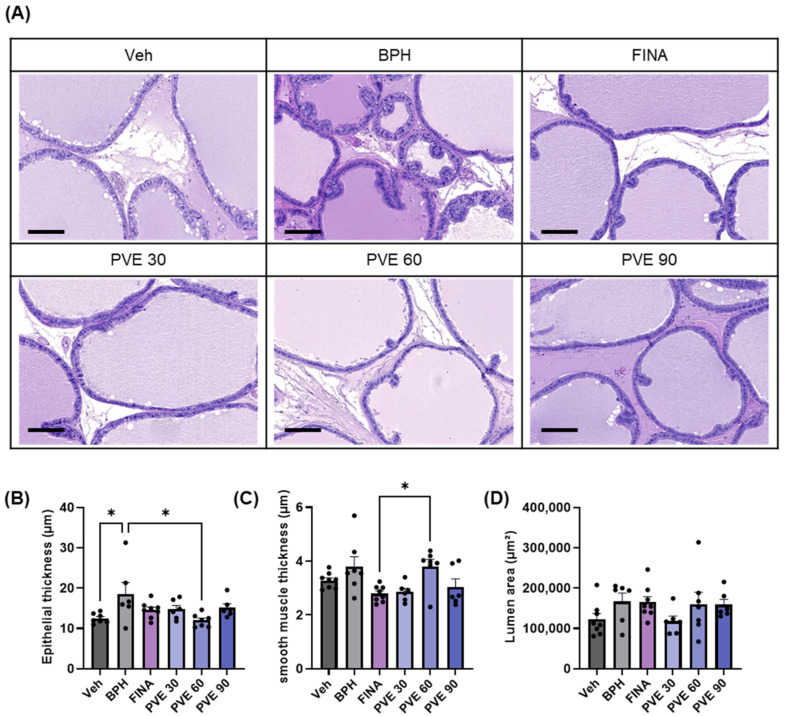
Histological analysis of the prostate (200× magnification, scale bar: 100 μm). (**A**–**C**) Summary of histological changes of epithelial thickness, smooth muscle thickness, and luminal area across groups. (**D**) Representative images of the prostate are shown. Data are shown as mean ± SEM. Statistical significance was evaluated using One-way ANOVA followed by Tukey’s multiple comparison test; * denotes *p* < 0.05 compared with TP-induced BPH prostate and the corresponding prostate response at each concentration, respectively.

The prostate was evaluated histologically by H&E staining to compare epithelial thickness, smooth muscle thickness, and lumen area. The epithelial thickness of the ventral prostate was significantly increased in the BPH group compared to the vehicle control group (Figure 5A,B). On the other hand, the group receiving PVE 60 mg/kg had a significant decrease in epithelial thickness compared to the BPH group (Figure 5B). Smooth muscle thickness was significantly different in the FINA and PVE 60 mg/kg groups, but there were no differences between the other groups, including PVE 30 and 90 (Figure 5C). The lumen area also showed no difference between groups (Figure 5D).

### 2.7. PVE Alleviates Bladder Dysfunction and Histologic Changes in In Vivo TP-Induced BPH Model

**Figure 6:**
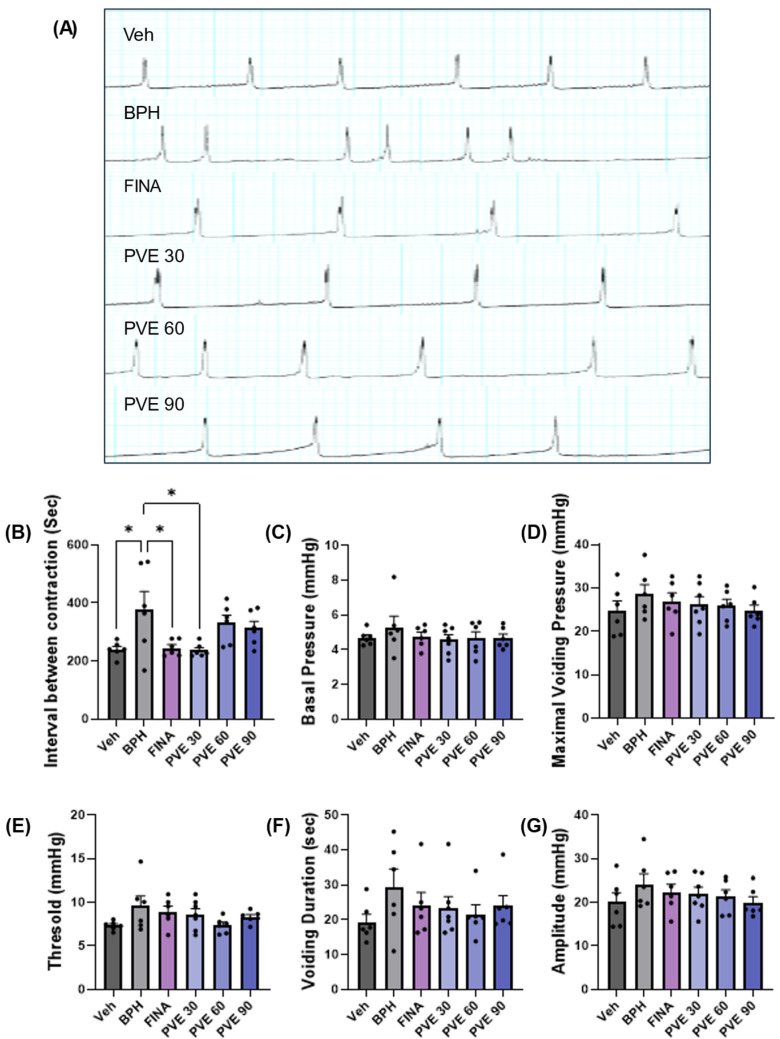
Effect of PVE on intravesical pressure in TP-induced BPH model. (**A**) Representitive image of the cystometry tracings from each group. (**B**) PVE improved the interval between contractions in the BPH model animal (* *p* < 0.05 vs. normal). (**C**–**G**) PVE was not associated with significant basal pressure, amplitude, threshold, and duration of voiding at any condition. Data are shown as mean ± SEM. Statistical significance was evaluated using one-way ANOVA followed by Tukey’s multiple comparison test.

Cystometry was performed in BPH model rats to determine whether PVE could improve BPH-induced LUTS. Figure 6A presents representative cystometry tracings from each group, depicting intravesical pressure changes during continuous filling (0.1 mL/min). The pattern of voiding changes with continuous infusion showed an irregular pattern in the BPH group. The interval between contraction time was statistically increased in the BPH group compared to the vehicle control group and was significantly improved by finasteride and PVE 30 mg/kg (Figure 6B). In contrast, there were no significant differences in basal pressure, maximal voiding pressure, threshold, voiding duration, or amplitude across all groups (Figure 6C–G).

**Figure 7:**
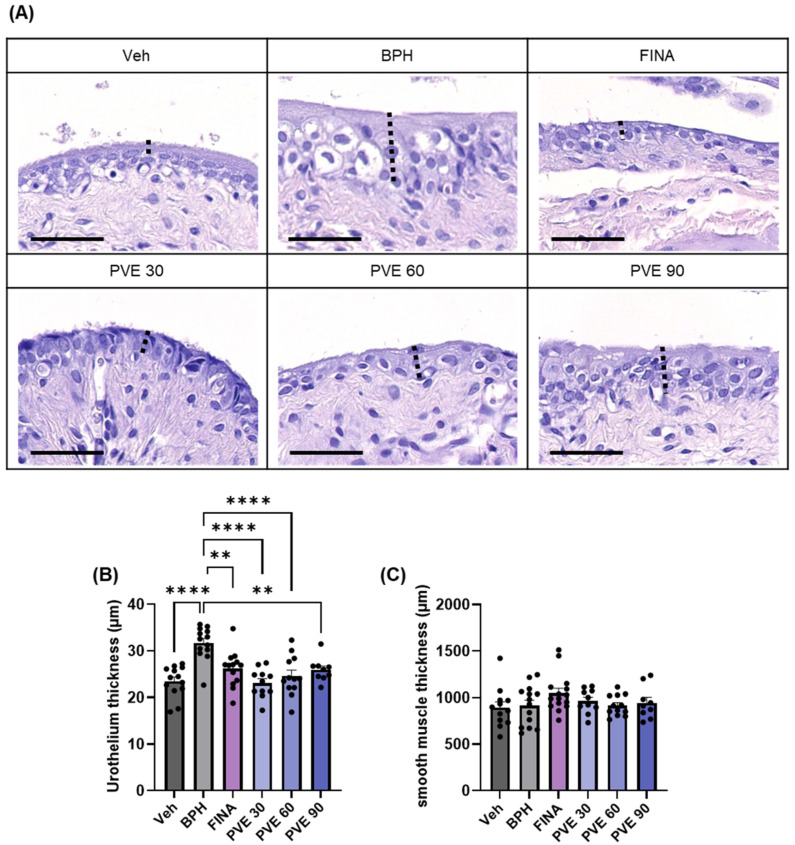
Effect of PVE on bladder histology in TP-induced BPH model. Representative H&E images of the urothelium of the bladder from different experimental groups (**A**–**C**) and the thickness of urothelium and smooth muscle were compared. Urinary bladder, 400× scale bar; 50 μm. Dashed line; Urothelium Data are shown as mean ± SEM. Statistical significance was evaluated using One-way ANOVA followed by Tukey’s multiple comparison test; ** *p* < 0.01 compared to the vehicle group and **** *p* < 0.0001 compared to the Finasteride group.

At the end of the experiment, the bladders were excised, stained with H&E, and evaluated histologically. The BPH group had significantly increased urothelial thickness than the NC group. Finasteride and PVE treatment significantly reduced urothelial thickness compared to the BPH group (Figure 7A,B). The thickness of bladder smooth muscle did not differ between treatment groups (Figure 7C).

## 3. Discussion

In the present study, we have shown that *Prunella vulgaris* extract exhibits concentration-dependent relaxation of prostatic smooth muscle. The effect of PVE is mediated by nitric oxide (NO) and voltage-gated potassium channels. PVE improved prostate hyperplasia in a TP-induced BPH rat model, reducing prostate weight and epithelial thickness and improving cystometric urodynamic parameters and bladder urothelial thickness caused by TP-induced BPH. Phytotherapy, which is well tolerated and has minimal side effects, is emerging as an alternative treatment for BPH/LUTS [14]. This study proposes PV as a candidate for phytotherapy. In this study, there was no significant change in the weight of the rats after four weeks of PVE administration, and no apparent side effects were observed. PV is generally considered non-toxic, with few reported side effects [16]. However, further toxicity assessment is necessary to validate its safety profile and support its clinical application.

α1-adrenergic antagonists are commonly used as a pharmacological treatment for lower urinary tract symptoms resulting from hypertrophy and contraction of prostate smooth muscle, facilitating prostate relaxation and improving clinical outcomes [30,31]. Various pharmacological approaches have been investigated to induce prostate smooth muscle relaxation, including thromboxane A2 antagonists [32], phosphodiesterase inhibitors [33], NOS activators [34], Kv7 channel modulators [35], and transient receptor potential cation (TRPC) channel inhibitors [36]. In the present study, PVE significantly inhibited PE-induced prostate smooth muscle contraction without affecting the concentration-response curve, suggesting a non-competitive mechanism. PVE-induced relaxation was significantly reduced by L-NAME and TEA, indicating the involvement of NO signaling and potassium channels. Furthermore, in order to determine which compounds of PVE are responsible for the prostate smooth muscle relaxation effect, we tested the effects of UA, which were identified as major compounds. However, UA did not have a significant effect on reducing PE-induced prostate smooth muscle contraction. We also screened candidate compounds to determine which compounds in PVE exerted a prostate smooth muscle relaxant effect. Quercetin, a polyphenol found in PV [29], was tested for its effects on prostate smooth muscle. Quercetin has been demonstrated to relax PE-induced contraction, consistent with the known properties of smooth muscle modulation and antioxidant activity [25,26]. Our qualitative HPLC analysis also confirmed the presence of quercetin in PVE used in this study. Therefore, the observed relaxation of prostate smooth muscle may be attributed to quercetin or other bioactive components. Since PV contains a variety of quercetin derivatives, including quercitrin, quercetin-3-O-glucoside, and quercetin-3-O-β-D-galactoside [16], further research is required to evaluate their potential effects on prostate smooth muscle. Additionally, to establish PV as a promising pharmaceutical candidate, it is crucial to develop an optimized extraction method that enhances the yield and consistency of bioactive flavonoids. PVE’s prostate smooth muscle relaxation via NO synthase activation and potassium channel modulation suggests its potential as a safe, effective natural therapy for BPH-related urinary symptoms.

5α-Reductase is an enzyme that plays a crucial role in the development of BPH [37]. The enlarged prostate in BPH causes lower urinary tract symptoms by obstructing the lower urinary tract, leading to urinary symptoms [37,38]. Therefore, control of prostate size through 5a-reductase inhibition is one of the important therapeutic interventions for BPH patients [39]. Previous studies [28] have confirmed that PVE efficiently suppresses prostate hyperplasia by regulating the proliferation and apoptosis of prostate tissue in a rat model of prostate hyperplasia. PVE extracted by the same method used in this study showed 5α-reductase inhibitory effects, leading to a significant reduction in prostate size being observed at low concentrations. This effect appears to affect prostate epithelial cells rather than prostate smooth muscle. In contrast, our study is the first to demonstrate that PVE directly inhibits ex vivo prostate smooth muscle contraction other than prostate epithelial cells and alleviates increased voiding intervals in BPH-induced SD rat models. Therefore, PV could be a potential natural product candidate for the treatment of BPH/LUTS. In order to accomplish this objective, it is imperative to establish a standardized extraction method for PV and subsequently conduct through qualitative and quantitative research to identify its active constituents.

In patients with BPH, the enlargement of the prostate gland can lead to increased pressure on the bladder. This chronic pressure can result in changes in the urothelium, including thickening, which is a common feature of BPH and can contribute to LUTS [40,41]. The thickened urothelium may alter sensory perception, leading to increased bladder sensitivity and urgency. This can result in frequent and urgent urination, as well as nocturia [42]. Also, the thickening of the urothelium can impair the normal contractile function of the detrusor muscle. This can lead to incomplete bladder emptying, a weak urine stream, and straining to urinate [41]. When considering the urothelium thickness, both finasteride and PVE can effectively reduce the thickening of the bladder lining, while no significant differences in bladder smooth muscle thickness were observed. This suggests that TP-induced BPH in rats does not significantly obstruct the lower urinary tract to induce detrusor hyperplasia, but it can induce urothelial damage and hyperplasia where PVE and finasteride act. Moreover, PVE, as a natural extract, has multiple mechanisms of action, including anti-inflammatory and antioxidant effects [16,43], which ameliorate urothelial damage at chronic high cystic pressure. However, its precise mode of action on bladder tissue is not fully understood. Our cystometric analysis showed an increased voiding interval in the BPH group, which was significantly reduced in the finasteride and PVE 30 groups. However, basal and maximal voiding pressures remained unchanged. Histologically, urothelial hypertrophic dysplasia was observed in the BPH group but not in the finasteride or PVE groups. While the direct clinical link between urothelial dysplasia and BPH remains unclear, it is frequently associated with obstructive voiding disorders and lower urinary tract symptoms [44]. The improvement in cystometric parameters and prevention of bladder epithelial thickening in this study supports the in vivo efficacy of PVE. Further research is needed to assess its direct impact on bladder tissue and therapeutic potential in other voiding disorder models beyond BPH.

The limitations of this study include: (1) Further research is needed to confirm the presence and quantity of various compounds in the PVE to ensure reproducibility and clarify the active compounds responsible for the effects. It is also necessary to establish an extraction method that maximizes the content of effective compounds. (2) The rat model of BPH, lacking urethral obstruction due to anatomical differences from the human prostate, resulted in less pronounced cystometric changes; and (3) the absence of direct urethral pressure measurements prevents a clear assessment of prostatic interference in urinary flow. These limitations hinder determining whether PVE primarily affects preventing bladder dysfunction.

## 4. Materials and Methods

### 4.1. Preparation of PV Extract

*Prunella vulgaris* L. (PV) was obtained from Jecheon Herbal Market (Jecheon, Chungbuk, Republic of Korea), and a voucher specimen (No. 2022-HF001, confirmed) was deposited at the College of Pharmacy, Han Yang University (Ansan, Republic of Korea). The PV was extracted using the maceration method, a widely used technique in medicinal plant research. This method was chosen due to its simplicity, cost-effectiveness, and efficiency in extracting flavonoids [45]. The PV extract was prepared by macerating dried PV spikes in 70% ethanol at 50 °C for 8 h, followed by a second extraction with 50% ethanol at 50 °C for 15 h. The combined extracts were concentrated under reduced pressure and lyophilized. The PV extract was manufactured by Huons Foodience under principles of Good Manufacturing Practice (GMP) standards and kept at −20 °C for further analysis.

### 4.2. HPLC Analysis

The extract (1 g) was dissolved in methanol in a 100 mL volumetric flask, filtered through a 0.45 μm PDVF filter, and analyzed using an Agilent 1260 series HPLC system (Agilent, Santa Clara, CA, USA) equipped with a diode array detector (DAD). The chromatographic separation was carried out on an Acclaim C30 column (250 × 4.6 mm, 5 μm) with the column temperature set at 25 °C, the detection wavelength at 210 nm, and the injection volume was 10 μL. An isocratic mobile phase consisting of methanol and 0.2% ammonium acetate was used at a flow rate of 0.4 mL/min. Standard of ursolic acid (UA, U6753) and quersetin (UA, Q4951) were purchased from Sigma Aldrich (Saint Louis, MO, USA). The compounds in the extract were identified by comparing their retention times and spectra with those of their respective standards.

### 4.3. Experimental Animals

Male Sprague Dawley rats (7 weeks old) were purchased from Samtako (Osan, South Korea). The animals were kept under standard laboratory settings (22 ± 2 °C, 50 ± 5% relative humidity, 12 h light/dark cycle) and fed rodent chow and sterilized tap water ad libitum. All animal protocols were approved by the Animal Experimental Ethics Committee of Chungnam National University (202404A-CNU, Daejeon, South Korea).

### 4.4. Preparation of Rat Prostate Smooth Muscle Strips and Measurement of PE-Induced Contraction

Male rats were anesthetized with carbon dioxide and euthanized by cervical dislocation. The midline incision was made in the abdomen, the lower urinary tract was isolated and immersed in cold physiological saline solution (120 mM/L NaCl, 2.5 mM/L CaCl_2_, 1 mM/L MgCl_2_, 11 mM/L Glucose, 25 mM/L NaHCO_3_, 5.9 mM/L KCl, and 1.2 mM/L NaH_2_PO_4_·H_2_O, pH 7.4). Fat and soft connective tissue were trimmed, and the prostate was separated. Only the ventral prostate was isolated from each lobe of the prostate. Longitudinal strips of prostate smooth muscle were made using a stereomicroscope (Nikon, Tokyo, Japan) in a silicon-coated petri dish containing a cold physiological saline solution. The prostate smooth muscle strips were connected using 6-0 silk sutures, knotted at each end with tungsten wire rings. One of the tungsten rings was then connected to the bottom of the organ bath and the other to a tension transducer (Grass Instruments, Quincy, MA, USA). The organ bath was filled with physiological saline solution at 37 °C and continuously supplied with 95% O_2_ and 5% CO_2_ gas to maintain physiological pH composition. PowerLab Data Acquisition system (ADInstruments, Sydney, Australia) and LabChart software (version 8) were used to measure contractions induced by PE, an α1-adrenergic receptor agonist. For the concentration-response curve of PE treatment, vehicle or PVE (300 μg/mL) was pretreated, and after 30 min, PE was added in increasing concentrations, and tension was recorded. Furthermore, to determine the smooth muscle relaxation effect of PVE in PE-induced contractions in prostate smooth muscle, contractions were induced with 10 μM of PE and treated with PVE in a concentration gradient. L-NAME (NOS inhibitor) and TEA (tetraethylammonium, K^+^ channel inhibitor) were also treated with PVE to explore the mechanism of PVE. The drugs used in the study were obtained from Sigma Aldrich (Saint Louis, MO, USA).

### 4.5. BPH Rat Model and PVE Administration

After a 1-week acclimatization period, a total of 50 rats were randomly assigned to one of the following groups: Veh (n = 8), BPH (n = 8), FINA (n = 8), PVE 30 (n = 8), PVE 60 (n = 8), and PVE 90 (n = 8). Stratified random sampling based on body weight was employed to ensure similar average weights across groups. The Veh group served as the vehicle control, without BPH induction, and received olive oil subcutaneously for 4 weeks along with PBS oral gavage. The BPH group received 3 mg/kg of testosterone propionate (TP) dissolved in olive oil daily for 4 weeks to induce BPH while receiving oral PBS. The FINA group underwent BPH induction in the same manner and received finasteride (10 mg/kg) orally each day for 4 weeks as a positive control. The PVE 30, PVE 60, and PVE 90 groups also induced BPH and received oral PVE doses of 30 mg/kg, 60 mg/kg, and 90 mg/kg, respectively. Daily for 4 weeks. Both PVE and finasteride were dissolved in phosphate-buffered saline (PBS), and the oral doses were administrated at a volume of 5 mL/kg. PVE was suspended in PBS, as it does not completely dissolve and tends to form a precipitate if left undisturbed. To ensure consistency and homogeneity, a fresh suspension was prepared daily, thoroughly mixed, and administered immediately via oral gavage.

### 4.6. Measurement of Intravesical Pressure

After the last administration, cystometry was performed to assess the voiding improvement effect of PVE. First, anesthesia was induced by administering urethane diluted in normal saline at 1.2 g/kg, SC, and leaving the animal still for 1 h. Once anesthesia was induced, rats were immobilized on a 37 °C constant temperature surgical table, and the incision site was de-furred and disinfected. The abdomen was opened using an inferior abdominal midline incision to expose the bladder. The dome of the exposed bladder was incised with an 18G needle to insert a polyethylene catheter (PE-50). The incision was then fixed by purse string suturing using non-absorbable nylon sutures (6-0). Using a three-way valve, the intubated PE catheter was connected to a manometer (Harvard appartus, Holliston, MA, USA) on one side and a syringe pump on the other side to infuse 0.9% normal saline at a constant rate of 100 μL/min. The measured intravesical pressure was recorded using a Powerlab (ADInstruments, Sydney, Australia) AD/DA converter and Labchart 8.0 (ADInstruments, Sydney, Australia), and analyzed for bladder function indicators of the interval between contractions, basal pressure, maximal voiding pressure, threshold potential, voiding duration, and amplitude.

### 4.7. Histology

After the last dose, the rats were sacrificed, and the bladder and prostate tissues were isolated and fixed in 10% buffered formalin. Following a 24-h fixation, the samples were dehydrated with a series of steps of EtOH and xylene and then embedded in paraffin to create paraffin embedding blocks. Paraffin blocks were sectioned at 4 μm thickness and mounted on glass slides for tissue staining. Sections were then deparaffinized in xylene and subjected to H&E staining with Harris hematoxylin solution (#H08-500R, TissuePro Technology, Hawthorne, FL, USA) and eosin Y solution (#EY07-500R, TissuePro Technology, Hawthorne, FL, USA). Stained tissue sections were scanned at ×400 magnification using an Aperio scan scope At (Leica Biosystems, Nussloch, Germany). Histologic evaluation of the scanned prostate and bladder was performed by an investigator blinded to group assignment using Image J software (version 46a; NIH, Bethesda, MD, USA). Prostate tissue was analyzed by measuring epithelial thickness, smooth muscle thickness, and lumen area in five random prostate acini from each rat and calculating the mean values. For bladder tissue, urothelium thickness and smooth muscle thickness were measured in five random areas for each rat, and the average value was calculated.

### 4.8. Statistical Analysis

All calculations and statistical analyses were performed using GraphPad Prism (version 8.4.2, GraphPad Software, Inc., San Diego, CA, USA). Statistical significance was assessed using one-way ANOVA followed by Tukey’s multiple comparison test. Data are presented as mean ± standard error of the mean (S.E.M.), with error bars in figures representing S.E.M. A *p*-value of <0.05 was considered statistically significant.

## 5. Conclusions

To summarize, our research revealed that PVE elicited a concentration-dependent relaxation of prostate smooth muscle by stimulating NO release and activating Kv channels and mitigated the lower urinary tract symptoms in a BPH rat model. Our findings demonstrated *Prunella vulgaris* L. (PV) has a potential therapeutic for BPH patients. However, further studies are needed to confirm its constituent substances and effectiveness in BPH patients.

## Data Availability

The datasets generated and analyzed during this study are available from the corresponding author (K.P.L.) upon reasonable request.

## Abbreviations

The following abbreviations are used in this manuscript:

BPH	benign prostate hyperplasia
LUTS	lower urinary tract symptoms
AR	androgen receptors
TP	testosterone propionate
HPLC	high-performance liquid chromatography
H&E	hematoxylin and eosin
α1-blockers	alpha1-adrenergic antagonists
DHT	dihydrotestosterone
PVE	*Prunella vulgaris* extract
UA	ursolic acid
PE	phenylephrine
NO	nitric oxide
NOS	nitric oxide synthase
L-NAME	Nω-nitro-L-arginine methyl ester hydrochloride
TEA	tetraethylammonium
TRPC	transient receptor potential cation

## References

[1] Berry S.J., Coffey D.S., Walsh P.C., Ewing L.L. (1984). The development of human benign prostatic hyperplasia with age. J. Urol..

[2] Bosch J., Tilling K., Bohnen A., Bangma C., Donovan J. (2007). Establishing normal reference ranges for prostate volume change with age in the population-based Krimpen-study: Prediction of future prostate volume in individual men. Prostate.

[3] McVary K.T., Roehrborn C.G., Avins A.L., Barry M.J., Bruskewitz R.C., Donnell R.F., Foster H.E., Gonzalez C.M., Kaplan S.A., Penson D.F. (2011). Update on AUA guideline on the management of benign prostatic hyperplasia. J. Urol..

[4] Bushman W. (2009). Etiology, epidemiology, and natural history. Urol. Clin..

[5] Fusco F., Creta M., De Nunzio C., Iacovelli V., Mangiapia F., Li Marzi V., Finazzi Agrò E. (2018). Progressive bladder remodeling due to bladder outlet obstruction: A systematic review of morphological and molecular evidences in humans. BMC Urol..

[6] Lepor H. (2005). Pathophysiology of lower urinary tract symptoms in the aging male population. Rev. Urol..

[7] Roehrborn C. (2008). Pathology of benign prostatic hyperplasia. Int. J. Impot. Res..

[8] McConnell J.D. (1991). The pathophysiology of benign prostatic hyperplasia. J. Androl..

[9] Foo K.T. (2017). Pathophysiology of clinical benign prostatic hyperplasia. Asian J. Urol..

[10] Lepor H. (2005). Pathophysiology of benign prostatic hyperplasia in the aging male population. Rev. Urol..

[11] Yu Z.J., Yan H.L., Xu F.H., Chao H.C., Deng L.H., Xu X.D., Huang J.B., Zeng T. (2020). Efficacy and Side Effects of Drugs Commonly Used for the Treatment of Lower Urinary Tract Symptoms Associated With Benign Prostatic Hyperplasia. Front. Pharmacol..

[12] Bortnick E.M., Simma-Chiang V., Kaplan S.A. (2019). Long-term Consequences of Medical Therapy for Benign Prostatic Hyperplasia. Rev. Urol..

[13] Berger A.P., Horninger W., Bektic J., Pelzer A., Spranger R., Bartsch G., Frauscher F. (2006). Vascular resistance in the prostate evaluated by colour Doppler ultrasonography: Is benign prostatic hyperplasia a vascular disease?. BJU Int..

[14] Antoniou V., Gauhar V., Modi S., Somani B.K. (2023). Role of Phytotherapy in the Management of BPH: A Summary of the Literature. J. Clin. Med..

[15] Rasool R., Ganai B.A. (2013). *Prunella vulgaris* L.: A literature review on its therapeutic potentials. Pharmacologia.

[16] Pan J., Wang H., Chen Y. (2022). *Prunella vulgaris* L.—A Review of its Ethnopharmacology, Phytochemistry, Quality Control and Pharmacological Effects. Front. Pharmacol..

[17] Psotová J., Kolář M., Soušek J., Švagera Z., Vičar J., Ulrichová J. (2003). Biological activities of *Prunella vulgaris* extract. Phytother. Res. Int. J. Devoted Pharmacol. Toxicol. Eval. Nat. Prod. Deriv..

[18] Fang X., Chang R.C.-C., Yuen W.-H., Zee S.Y. (2005). Immune modulatory effects of *Prunella vulgaris* L.. Int. J. Mol. Med..

[19] Li C., Huang Q., Fu X., Yue X.-J., Liu R.H., You L.-J. (2015). Characterization, antioxidant and immunomodulatory activities of polysaccharides from *Prunella vulgaris* Linn. Int. J. Biol. Macromol..

[20] Wang S.-J., Wang X.-H., Dai Y.-Y., Ma M.-H., Rahman K., Nian H., Zhang H. (2019). *Prunella vulgaris*: A comprehensive review of chemical constituents, pharmacological effects and clinical applications. Curr. Pharm. Des..

[21] Gu X., Li Y., Mu J., Zhang Y. (2013). Chemical constituents of *Prunella vulgaris*. J. Environ. Sci..

[22] Somova L., Nadar A., Rammanan P., Shode F. (2003). Cardiovascular, antihyperlipidemic and antioxidant effects of oleanolic and ursolic acids in experimental hypertension. Phytomedicine.

[23] Tsai S.J., Yin M.C. (2008). Antioxidative and anti-inflammatory protection of oleanolic acid and ursolic acid in PC12 cells. J. Food Sci..

[24] Feng L., Jia X., Zhu M.-M., Chen Y., Shi F. (2010). Antioxidant activities of total phenols of *Prunella vulgaris* L. in vitro and in tumor-bearing mice. Molecules.

[25] Choi Y.-J., Fan M., Wedamulla N.E., Tang Y., Kim E.-K. (2024). Alleviatory effect of isoquercetin on benign prostatic hyperplasia via IGF-1/PI3K/Akt/mTOR pathway. Food Sci. Hum. Wellness.

[26] Ghorbanibirgani A. (2012). Efficacy of quercetin in treatment of benign prostatic hyperplasia in a double-blind randomized clinical trial in Iran 2011. Contraception.

[27] Huang N., Hauck C., Yum M.-Y., Rizshsky L., Widrlechner M.P., McCoy J.-A., Murphy P.A., Dixon P.M., Nikolau B.J., Birt D.F. (2009). Rosmarinic acid in *Prunella vulgaris* ethanol extract inhibits lipopolysaccharide-induced prostaglandin E2 and nitric oxide in RAW 264.7 mouse macrophages. J. Agric. Food Chem..

[28] Kumbukgahadeniya P., Baek E.-B., Hong E.-J., Song J.-Y., Kwak Y.-G., Jang M.-R., Ji H.-S., Kwun H.-J. (2024). *Prunella vulgaris* Extract Ameliorates Testosterone-Induced Benign Prostatic Hyperplasia by Regulating Androgen Levels, Cell Proliferation, and Apoptosis. Pharmaceuticals.

[29] Deng J., Li L., Yan J., Lin L.-M., Li Y.-M., Lin Y., Xia B.-H. (2022). Essential oil from *Prunella vulgaris* L. as a valuable source of bioactive constituents: In vitro anti-bacterial, anti-viral, immunoregulatory, anti-inflammatory, and chemical profiles. S. Afr. J. Bot..

[30] Wang Y., Kunit T., Ciotkowska A., Rutz B., Schreiber A., Strittmatter F., Waidelich R., Liu C., Stief C., Gratzke C. (2015). Inhibition of prostate smooth muscle contraction and prostate stromal cell growth by the inhibitors of R ac, NSC 23766 and EHT 1864. Br. J. Pharmacol..

[31] Spek A., Li B., Rutz B., Ciotkowska A., Huang R., Liu Y., Wang R., Strittmatter F., Waidelich R., Stief C.G. (2021). Purinergic smooth muscle contractions in the human prostate: Estimation of relevance and characterization of different agonists. Naunyn-Schmiedeberg’s Arch. Pharmacol..

[32] Hennenberg M., Tamalunas A., Wang Y., Keller P., Schott M., Strittmatter F., Herlemann A., Yu Q., Rutz B., Ciotkowska A. (2017). Inhibition of agonist-induced smooth muscle contraction by picotamide in the male human lower urinary tract outflow region. Eur. J. Pharmacol..

[33] Hennenberg M., Schott M., Kan A., Keller P., Tamalunas A., Ciotkowska A., Rutz B., Wang Y., Strittmatter F., Herlemann A. (2016). Inhibition of Adrenergic and Non-Adrenergic Smooth Muscle Contraction in the Human Prostate by the Phosphodiesterase 10-Selective Inhibitor TC-E 5005. Prostate.

[34] Calmasini F.B., Silva F.H., Alexandre E.C., Rodrigues R.L., Barbosa A.P.L., Ferrucci D.L., Carvalho H.F., Anhê G.F., Pupo A.S., Antunes E. (2017). Implication of Rho-kinase and soluble guanylyl cyclase enzymes in prostate smooth muscle dysfunction in middle-aged rats. Neurourol. Urodyn..

[35] Choi J., Han D.H., Chae M.R., Sung H.H., Kang S.J., Shin J., Lee S.W. (2021). Molecular expression and functional features of Kv7 channels in human prostate smooth muscle. Andrology.

[36] Thébault S., Zholos A., Enfissi A., Slomianny C., Dewailly E., Roudbaraki M., Parys J., Prevarskaya N. (2005). Receptor-operated Ca2+ entry mediated by TRPC3/TRPC6 proteins in rat prostate smooth muscle (PS1) cell line. J. Cell. Physiol..

[37] Asiedu B., Anang Y., Nyarko A., Doku D.A., Amoah B.Y., Santa S., Ngala R.A., Asare G.A. (2017). The role of sex steroid hormones in benign prostatic hyperplasia. Aging Male.

[38] Carson III C., Rittmaster R. (2003). The role of dihydrotestosterone in benign prostatic hyperplasia. Urology.

[39] Madersbacher S., Sampson N., Culig Z. (2019). Pathophysiology of benign prostatic hyperplasia and benign prostatic enlargement: A mini-review. Gerontology.

[40] Chapple C.R. (2019). Moving beyond BPH–A contemporary update on male LUTS. Asian J. Urol..

[41] Novara G., Galfano A., Gardi M., Ficarra V., Boccon-Gibod L., Artibani W. (2006). Critical review of guidelines for BPH diagnosis and treatment strategy. Eur. Urol. Suppl..

[42] Salah Azab S., Elsheikh M.G. (2015). The impact of the bladder wall thickness on the outcome of the medical treatment using alpha-blocker of BPH patients with LUTS. Aging Male.

[43] Zhang M., Hwang E., Lin P., Gao W., Ngo H.T., Yi T.-H. (2018). *Prunella vulgaris* L. exerts a protective effect against extrinsic aging through NF-κB, MAPKs, AP-1, and TGF-β/Smad signaling pathways in UVB-aged normal human dermal fibroblasts. Rejuvenation Res..

[44] Dunton C.L., Purves J.T., Hughes F.M., Nagatomi J. (2021). BOO induces fibrosis and EMT in urothelial cells which can be recapitulated in vitro through elevated storage and voiding pressure cycles. Int. Urol. Nephrol..

[45] Subramanian P., Anandharamakrishnan C. (2023). Extraction of bioactive compounds. Industrial Application of Functional Foods, Ingredients and Nutraceuticals.

